# Lead-Related Infective Endocarditis in Latvia: A Single Centre Experience

**DOI:** 10.3390/medicina55090566

**Published:** 2019-09-03

**Authors:** Nikolajs Nesterovics, Georgijs Nesterovics, Peteris Stradins, Martins Kalejs, Janis Ansabergs, Maris Blumbergs, Aija Maca, Ginta Kamzola, Aivars Lejnieks, Oskars Kalejs, Andrejs Erglis

**Affiliations:** 1 Latvian Centre of Cardiology, Pauls Stradins Clinical University Hospital, LV-1002 Riga, Latvia; 2 Faculty of Medicine, Riga Stradins University, LV-1007 Riga, Latvia; 3 Faculty of Medicine, University of Latvia, LV-1004 Riga, Latvia; 4 Department of Endocrinology, Riga East University Hospital, LV-1038 Riga, Latvia

**Keywords:** lead-related infective endocarditis, cardiac implantable electronic devices, cardiac device infection, CIED complication, LRIE mortality

## Abstract

*Background and Objectives*: Over the last five decades cardiac implantable electronic devices (CIED) have become established as the mainstay for the treatment of permanent bradycardias, chronic heart failure and dangerous heart rhythm disturbances. These devices improve survival and quality of life in many patients. However, infections associated with CIED implantation, particularly lead-related infective endocarditis (LRIE), can offset all benefits and make more harm than good for the patient. To date, there are no other studies in Latvia, addressing patients with lead-related infective endocarditis. The objective of this study was to identify the most common pathogens associated with LRIE and their antimicrobial resistance and to identify possible risk factors of patients who present with LRIE. *Materials and Methods*: The study was performed retrospectively at Pauls Stradins Clinical University Hospital (PSCUH). The study included patients who were referred to PSCUH due to LRIE for lead extraction. Patients were identified from procedural journals. Information about isolated microorganisms, patient comorbidities and visual diagnostics data was taken from patient records. *Results*: Forty-nine patients with CIED related infective endocarditis were included in the study, 34 (69.4%) were male, median age of all patients was 65.0 (50.5–73.0) years, median hospital stay was 15.5 (22.0–30.5) days. Successful and complete lead extraction was achieved in all patients. Thirty-two (65.3%) had received antibiotics prior to blood sample. Only in 31 (63.3%) positive culture results were seen. The most common isolated pathogens were *Staphylococcus aureus* (23.5%) and coagulase negative staphylococci (23.5%). Other bacteria were isolated considerably less often. The atrial lead was most common location for lead vegetations, seen in 50.0% of cases. Five (10.2%) patients have died due to the disease. *Conclusions*: Lead-related infective endocarditis is a major complication of cardiac implantable electronic devices with considerable morbidity and mortality, which in our study was as high as 10.2%.

## 1. Introduction

Cardiac implantable electronic devices (CIED), which include permanent pacemakers, cardiac resynchronization therapy (CRT) and implantable cardioverter defibrillators (ICD), over the past five decades have become established as a mainstay for the treatment of many permanent bradycardias and other conduction delays, chronic heart failure and various dangerous heart rhythm disturbances. Due to aging population, good medicine standards and better patient management, a lot of patients survive serious cardiac events and live longer with more significant cardiac disease and other comorbidities, which means that the number of patients with CIED or who require a CIED is significantly increasing every year [1]. Recent data suggest that at least 1.2 million devices are implanted annually [2].

By optimizing cardiac conduction and preventing sudden cardiac death, these devices further improve survival and quality of life in many individuals. However, infections associated with CIED implantation, particularly lead-related infective endocarditis (LRIE), can offset all benefits and lead to grave consequences. Nearly all these patients have to undergo a complex surgical procedure with complete lead and generator removal, with following prolonged hospitalization and massive antibacterial treatment. Unfortunately, despite all these measures, mortality associated with LRIE remains high. In a retrospective study at Mayo Clinic, which had covered a time period between years 1991 and 2008, 30-day in-hospital intrahospital mortality was 2–5% [3], but one-year mortality can be as high as 17–20% [3,4,5].

Unfortunately, despite the growing experience with device implantation, the rate of CIED infection has been increasing in recent years. This can be partly explained by increasing age and morbidity of device recipients, as well as by increasing rate of implantation of more complex devices, such as CRT and CRT defibrillators (CRT-D).

The diagnosis of LRIE is frequently delayed, due to non-specific clinical signs, such as pyrexia, shortness of breath and malaise, as well as low suspicion of LRIE among general practitioners. In a study by Klug D. and his colleagues, fever occurred in 86.5% of 52 patients, elevated C-reactive protein was found in 96.2% and clinical and/or radiological evidence of pulmonary involvement were seen in 38.4% of patients. In general, CIED infection should be suspected and ruled out in all patients with CIED and otherwise unexplained fever [6,7].

There are many known risk factors which predispose an individual with CIED to a device related infection. Diabetes mellitus, renal dysfunction (defined as glomerular filtration rate (GFR) < 60 mL/min/1.72 m^2^) and heart failure are most frequent comorbidities of patients who present with CIED infections. Long term corticosteroid and/or anticoagulant use are also associated with higher infection risk in these patients [7,8,9]. Certain procedural characteristics can also increase the risk—such as low operator experience, fewer within 24 h before implantation, the use of temporary pacing before device implantation and early reintervention. Generator replacement, rather than primary implantation, carries greater risk for device infection [7,10].

Identification of causative organism and its antimicrobial resistance is a crucial part of management of LRIE. This is usually done by cultures from blood, removed lead fragments and from pocket material. There are two ways a causative organism can infect the pocket and leads. The pocket can become infected at the time of implantation or replacement of device/generator. The organism then spreads along the electrode and infects intracardiac structures. The alternative way is hematogenous spread of the organism from the distant secondary site of infection. Many species of bacteria, after adhering to the lead, will form an extracellular polymer matrix and engulf themselves in so called biofilm, which makes the bacteria highly resistant to host defense mechanisms and antibacterial treatment. For this reason, identification of causative bacterium, extraction of infected device, followed by effective antibacterial treatment are cornerstones in management of lead-related infective endocarditis [2].

Number of implanted devices in Latvia is increasing and in the last couple of years approximately up to 1500 devices were implanted per year. There are two hospitals where these procedures are performed—Pauls Stradins Clinical University Hospital and Riga East University Hospital. All kinds of devices are used accordingly to every individual patient indication—pacemakers, ICDs, CRTs, CRT-Ds. However, despite the growing implantation numbers, to this date, there are no studies addressing LRIE causal microorganisms and its associated risk factors in Latvia. The aim of this study was to identify the most commonly isolated pathogens and their antimicrobial resistance data, as well as the possible risk factors of patients who present LRIE. The study will allow to compare the causal microorganisms with those from other countries and to estimate the antimicrobial resistance rate of LRIE causal bacteria in Latvia.

## 2. Materials and Methods

This was a retrospective study, performed at Pauls Stradins Clinical University Hospital (PSCUH). Study included patients, who were referred to PSCUH due to lead-related infective endocarditis, for lead extraction during a time period between 1 January 2000 and 31 December 2016. Patients who presented local pocket infection but without lead-related infective endocarditis were not included in this study.

At PSCUH, the choice of therapeutic approach to the patient with LRIE depends on patient’s clinical status (e.g., hemodynamic parameters and number/seriousness of comorbidities) and extent of lead and/or valve involvement. There are two main approaches to lead extraction—transvenous and surgical. If patient is fit for surgery and/or has valve involvement, surgical approach is chosen, while if patient is not fit for surgery transvenous approach is considered. If patient undergoes transvenous lead extraction, temporary transvenous pacing system is used together with antibiotic therapy for at least 7 days or longer—until inflammatory markers become acceptable for implantation of next permanent system. In cases, when surgical approach is chosen, after lead extraction is completed, during the same surgical procedure, a new system with epicardial leads is implanted on the opposite side, with following antibacterial therapy as appropriate.

Causal microorganisms were cultivated from blood, lead fragments and from the device pocket. LRIE was considered and reported as culture-negative if results from all culture locations were negative. Information about patients transthoracic (TTE) or transesophageal echocardiographic (TOE) exams was recorded. If a patient underwent both types of echocardiography, only TOE results were considered. Patients comorbidities were also recorded. Patients were identified from procedural journals, where records of lead and device extraction were found. There were no age restrictions in this study.

IBM SPSS v22.0 software was used for data analysis. If normally distributed, continuous variables were shown as the mean and standard deviation and as median and inter-quartile range if there is non-normal distribution. Student’s *t*-test was used for normally distributed continuous variables, while Mann–Whitney U test was used for non-normally distributed continuous variables. Chi-square or Fisher’s tests were used for comparison of categorical variables as appropriate. Statistically significant results were defined as results with *p* value of <0.05.

The study was approved by Pauls Stradins Clinical University Hospital Ethics committee on 28 May 2019. Study approval number: 280519-34 L. All data were collected in accordance with the standards of the Riga Stradins University.

## 3. Results

We have identified 49 patients with CIED lead-related endocarditis who were referred to PSCUH for lead extraction in time period between 1 January 2000 and 31 December 2019. Thirty-four (69.4%) were male; median age of all patients was 65.0 (50.5–73.0) years. During the study period a total number of implanted CIED was around 18,150 devices—LRIE developed in 0.27% of our patients with implanted devices.

Twenty-six patients (53.1%) prior to development of LRIE had device upgrade or generator replacement or lead replacement, while 23 (46.9%) developed the disease after primary CIED implantation. Precise date of previous manipulation with the device was known in 33 (67.3%) patients. Median time since implantation/reimplantation in these patients was 246.0 (88.0—1175.5) days. In the rest of cases, only year of manipulation was known. From known data, it can be deduced that 28 (57.1%) patients developed LRIE within two years after the last manipulation.

Forty-one patients (83.7%) had permanent pacemaker, 7 (14.3%) had CRT or CRT-D and 1 (2.0%) had ICD. Two lead devices were seen in 36 (73.5%) patients, while one and three lead devices in 6 (12.2%) and 7 (14.3%) cases, respectively. There was a total of 99 leads in our study. Surgical lead extraction was carried out in 34 (69.4%) patients, while transvenous approach was chosen in 15 (30.6%). In all cases a complete and successful lead extraction was achieved.

Median number of days spent in hospital was 15.5 (22.0–30.5). Hospital stay was not statistically significantly affected by age (r_s_ = 0.2, *p* = 0.131), gender (*p* = 0.274) or type of lead extraction (*p* = 0.948).

### 3.1. Pathogens Associated with Lead-Related Infective Endocarditis

**Figure 1:**
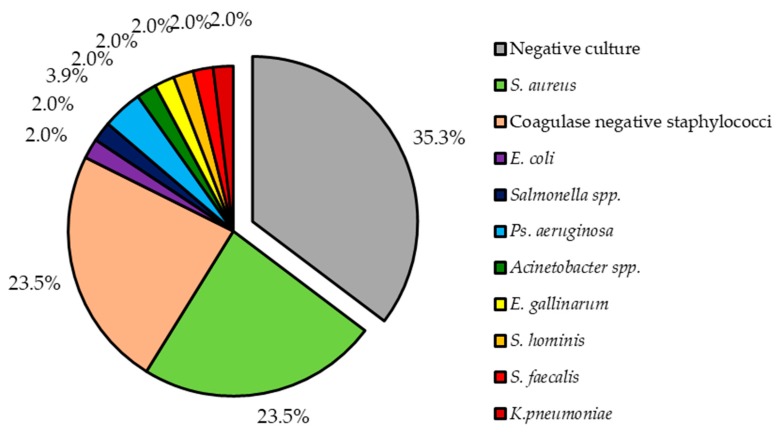
Pathogens isolated from patients with lead-related infective endocarditis.

Out of 49 patients, 32 (65.3%) have received antibiotics prior to blood sample. As the result, only in 31 (63.3%) positive culture results were seen. Two (4.1%) patients had infection caused by two bacteria (in the first case multi-infection caused by *S. aureus* and *Pseudomonas aeruginosa* and in the second case infection caused by *Enterococcus faecalis* and *Klebsiella pneumoniae*), while all others by a single bacterium. Two most common bacteria types isolated from our patients were *S. aureus* and coagulase negative staphylococci (CoNS). Together both of these accounted for 47.0% (*n* = 24), each for 23.5% (*n* = 12). Other isolated bacteria species are shown in Figure 1.

### 3.2. Antimicrobial Resistance of Isolated Pathogens

When analyzing the resistance of all bacteria at once, Penicillin G had the highest resistance rate out of all tested antimicrobial agents—in 75.0% of tested cases the resistance to its action was seen—while the second highest resistance was to Erythromycin—25.0% of tested cases were resistant to this drug. The least resistance rate was to Linezolid (0.0%), Tetracycline (5.0%) and Trimethoprim/Sulfamethoxazole (7.7%). The resistance to Gentamycin, which is frequently used in treatment of endocarditis in combination with Vancomycin, was 11.1%. None of the isolates was resistant to Vancomycin, however, resistance to its action was tested only in 12.1% bacteria. Resistance to most frequently tested drugs can be seen in Table 1. The resistance to less frequently tested drugs will not be reported in this article.

From the perspective of most commonly isolated bacteria, *S. aureus* was resistant to at least one antimicrobial agent in 54.5% (*n* = 6) of cases. In one case, there were no antimicrobial resistance data available for *S*. *aureus*. High susceptibility was seen to many antimicrobial drugs—100.0% nearly in all cases. However, 70.0% of these isolates were resistant to Penicillin G. Whole resistance results of *S. aureus* can be seen in the Table A1.

Coagulase negative staphylococci were resistant in 83.3% (*n* = 10) of cases to at least one agent. Highest resistance was seen to Penicillin G (71.4%), Erythromycin (45.5%) and to Trimethoprim/Sulfamethoxazole (33.3%). Low resistance was seen to Gentamycin (0.0%). Whole resistance results of *S. aureus* can be seen in the Table A2. In general, CoNS were more resistant then *S. aureus*. The resistance of other bacteria will not be analyzed in this article due to their low numbers.

### 3.3. Echocardiographic Findings in Patients with LRIE

**Figure 2:**
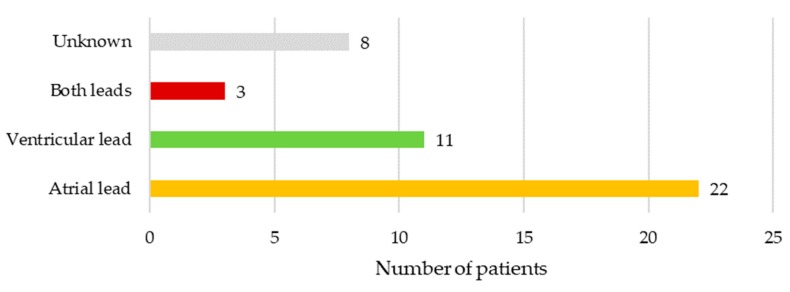
Location of vegetations in patients with lead-related infective endocarditis.

In 10.2% (*n* = 5) of cases, there were no vegetations seen on leads or heart valves on either transthoracic or transesophageal echocardiography exam. In another 10.2% (*n* = 5) of cases vegetations were seen on leads and valves, while in 79.6% (*n* = 39) vegetations were only on device leads. Only atrial lead was affected in 22 (50.0%), only ventricular lead in 11 (25.0%), while both leads were infected in 3 (6.8%) patients. In 8 (18.2%) cases the precise location of vegetations on either atrial and/or ventricular lead was not specified. Location of vegetations is illustrated in Figure 2.

### 3.4. Comorbidities and Mortality Associated with LRIE

Most frequent comorbidities of patients, who developed LRIE were: Chronic heart failure 83.7% (*n* = 41) (NYHA I-II 56.1% (*n* = 23), NYHA III-IV 43.9% (*n* = 18)), coronary heart disease 57.1% (*n* = 28), chronic kidney disease 22.4% (*n* = 11), diabetes mellitus 18.4% (*n* = 9), gout 10.2% (*n* = 5) and 4.1% (*n* = 2) had active oncology. Eight (16.3%) patients had other artificial body implants (e.g., artificial heart valves or hip prothesis) and eight (16.3%) had major cardiac surgery in anamnesis. Five (10.2%) patients have died due to LRIE.

## 4. Discussion

Lead-related infective endocarditis is a rare serious complication of cardiac electronic implantable devices with non-specific early signs, which is associated serious consequences and high mortality. This is a first insight in LRIE experience in Latvia. We have identified 49 patients with LRIE, out of whom 5 (10.2%) patients have died due to the disease. Mortality rate was similar to data reported in other studies [11]. Relatively low number of patients with LRIE in Latvia can be explained by high experience of operators who perform these procedures. There are two hospitals in Latvia where all these devices are implanted. As there are few operators who implant these devices, most of them perform >300 procedures per year. Prevention of CIED infection during the implantation of these devices is briefly described in the Appendix B. It is also possible that a few patients were not fit for lead extraction at all, in which case there are no records of these patients in the procedure journals and therefore they were not identified by us in this study. Due to the fact that there is no unique disease code for LRIE, it is nearly impossible to identify patients with LRIE in retrospective setting in any other way.

In our experience, LRIE more frequently developed after reimplantation, rather than after primary implantation, which can be supported by some other, bigger LRIE studies [12]. More than a half of patients (57.1%) in our study have developed the disease within two years of previous invasive manipulation of the implanted device. Identification of causal microorganism in patients with LRIE can sometimes be impossible due to prior antibiotic treatment. Due to low suspicion of LRIE and non-specific early clinical sings, such as shortness of breath and pyrexia, physicians frequently prescribe antibiotics for these patients weeks before the correct diagnosis is made [7]. For this reason, in our study 14 (43.8%) out of 32 patients, who have received antibiotics, had negative bacterial culture findings. In the patient group with positive cultures, two most commonly isolated bacteria were *S. aureus* and coagulase negative staphylococci, which is similar to causal flora in many other LRIE studies and reports [5,7,11,13]. *S. aureus* isolates were highly resistant to Penicillin G—in up to 70.0% of cases. However, there was good susceptibility to all other tested antimicrobials, such as Gentamycin. In the meantime, CoNS showed considerably higher resistance rates than *S. aureus*. The possible explanation is that infection with *S. aureus* was acquired in the community setting, while infection with coagulase negative staphylococcic was healthcare related. In consideration of known data and of the resistance rate of all isolated pathogens, empirical treatment with combination of Gentamycin with Vancomycin seems appropriate until a causal microorganism with its antimicrobial resistance is identified. Identification of the disease was further complicated by the fact that in 10.2% of cases there were no clear echocardiographic signs of vegetations on leads or heart valves.

Most frequent risk factors associated with LRIE in our patients were diabetes mellitus and chronic kidney disease, which goes well along with other studies [8,9]. It is rather interesting that 10.2% of patients had gout as a comorbidity, which is at least 5 times more than it is seen in general population. In some patients it could develop secondary to chronic kidney disease, but this is also possible due to the small population in the study.

This study is a first study of LRIE in Latvia, which reports causal microorganisms, antimicrobial resistance and disease outcomes. Disease rarity and low patient population are major limitations of this study. Another important limitation is its retrospective nature—a lot of data is nearly impossible to acquire in this setting. A long-term prospective study is needed for a better understanding of LRIE. However, this study is a first good insight into LRIE in Latvia, which will allow us to study the disease further and improve the knowledge, understanding and awareness of the disease among other specialists.

## 5. Conclusions

LRIE is a major complication of CIED with high morbidity and mortality. Our reported data and patients are in many ways similar to other studies. Two most commonly isolated LRIE causal pathogens were *S. aureus* and coagulase negative staphylococci. Both organisms frequently showed considerable antimicrobial resistance to Penicillin G. *Staphylococcus aureus*, in general, was less resistant to antimicrobials than CoNS. Empirical treatment with combination of Gentamycin and Vancomycin seem appropriate in the empirical setting,

## Figures and Tables

**Table 1:** The resistance to most frequently tested antimicrobial agents.

Title of the Antimicrobial Agent	Resistance ^a^	Times Tested ^b^
Ciprofloxacin	4 (14.8)	27
Gentamycin	3 (11.1)	27
Erythromycin	6 (25.0)	24
Clindamycin	3 (13.0)	23
Rifampicin	2 (8.7)	23
Penicillin G	15 (75.0)	20
Tetracycline	1 (5.0)	20
Chloramphenicol	2 (11.1)	18
Linezolid	0 (0.0)	13
Trimethoprim/Sulfamethoxazole	1 (7.7)	13
Oxacillin	0 (0.0)	7
Ceftazidime	2 (40.0)	5
Imipenem	0 (0.0)	5
Piperacillin/Tazobactam	0 (0.0)	5
Ampicillin	1 (25.0)	4
Vancomycin	0 (0.0)	4

^a^ Number of resistant strains (%); ^b^ number of times, when the resistance to specific antimicrobial agent was tested.

## Appendix A

**Table A1:** The resistance of *S. aureus* to most frequently tested antimicrobial agents.

Title of the Antimicrobial Agent	Resistance ^a^	Times Tested ^b^
Ciprofloxacin	1 (9.1)	11
Gentamycin	0 (0.0)	11
Erythromycin	0 (0.0)	10
Clindamycin	0 (0.0)	10
Trimethoprim/Sulfamethoxazole	0 (0.0)	10
Penicillin G	7 (70.0)	10
Tetracycline	0 (0.0)	9
Rifampicin	0 (0.0)	9
Chloramphenicol	0 (0.0)	7
Linezolid	0 (0.0)	7
Oxacillin	0 (0.0)	5

^a^ Number of resistant strains (%); ^b^ number of times, when the resistance to specific antimicrobial agent was tested.

**Table A2:** The resistance of coagulase negative staphylococci to most frequently tested antimicrobial agents.

Title of the Antimicrobial Agent	Resistance ^a^	Times Tested ^b^
Erythromycin	5 (45.5)	11
Clindamycin	2 (18.2)	11
Gentamycin	0 (0.0)	9
Ciprofloxacin	1 (11.1)	9
Rifampicin	1 (11.1)	9
Tetracycline	1 (11.1)	9
Trimethoprim/Sulfamethoxazole	3 (33.3)	9
Chloramphenicol	1 (14.3)	7
Penicillin G	5 (71.4)	7
Linezolid	0 (0.0)	4

^a^ Number of resistant strains (%); ^b^ number of times, when the resistance to specific antimicrobial agent was tested.

## Appendix B

The aspects of prevention of CIED infection in our center are reported here:

- There is a special procedural room in PSCUH, which is used only for CIED implantations. This room undergoes a regular cleaning and ultraviolet disinfection every day.
- Number of people in the procedural room is strictly limited to the patient, operator and surgical nurse.
- In general, if patient has elevated inflammatory markers, CIED implantation is delayed until these markers become acceptable.
- Antimicrobial prophylaxis with parenterally administered Cefazolin 1 h before the implantation is used in all patients.
- Preprocedural skin antisepsis is achieved with propanol-based antiseptic.
- Incise drapes are used during implantation.
- Antibacterial envelopes for CIED are available and used on rare occasions.
- After the procedure ice compression is used to achieve better hemostasis.
- Patients are discharged only on the next day after the procedure.
